# Performance of a targeted cell‐free DNA prenatal test for 22q11.2 deletion in a large clinical cohort

**DOI:** 10.1002/uog.23699

**Published:** 2021-10-01

**Authors:** E. Bevilacqua, J. C. Jani, R. Chaoui, E.‐K. A. Suk, R. Palma‐Dias, T.‐M. Ko, S. Warsof, R. Stokowski, K. J. Jones, F. R. Grati, M. Schmid

**Affiliations:** ^1^ Department of Obstetrics and Gynecology, University Hospital Brugmann Université Libre de Bruxelles Brussels Belgium; ^2^ Prenatal Diagnosis and Human Genetics Berlin Germany; ^3^ The Royal Women's Hospital University of Melbourne Parkville VIC Australia; ^4^ Genephile Bioscience Laboratory, Ko's Obstetrics & Gynecology Clinic Taipei City Taiwan; ^5^ Eastern Virginia Medical School Norfolk VA USA; ^6^ Roche Sequencing Solutions, Inc. San Jose CA USA; ^7^ TOMA Advanced Biomedical Assays S.p.A, Impact Lab. Busto Arsizio Italy

**Keywords:** 22q11.2 deletion, cell‐free DNA, DiGeorge syndrome, microdeletion, NIPT, prenatal diagnosis

## Abstract

**Objective:**

22q11.2 deletion is more common than trisomies 18 and 13 combined, yet no routine approach to prenatal screening for this microdeletion has been established. This study evaluated the clinical sensitivity and specificity of a targeted cell‐free DNA (cfDNA) test to screen for fetal 22q11.2 deletion in a large cohort, using blinded analysis of prospectively enrolled pregnancies and stored clinical samples.

**Methods:**

In order to ensure that the analysis included a meaningful number of cases with fetal 22q11.2 deletion, maternal plasma samples were obtained by prospective, multicenter enrolment of pregnancies with a fetal cardiac abnormality and from stored clinical samples from a research sample bank. Fetal genetic status, as evaluated by microarray analysis, karyotyping with fluorescence *in‐situ* hybridization or a comparable test, was available for all cases. Samples were processed as described previously for the Harmony prenatal test, with the addition of DANSR (Digital Analysis of Selected Regions) assays targeting the 3.0‐Mb region of 22q11.2 associated with 22q11.2 deletion syndrome. Operators were blinded to fetal genetic status. Sensitivity and specificity of the cfDNA test for 22q11.2 deletion were calculated based on concordance between the cfDNA result and fetal genotype.

**Results:**

The final study group consisted of 735 clinical samples, including 358 from prospectively enrolled pregnancies and 377 stored clinical samples. Of 46 maternal plasma samples from pregnancies with a 22q11.2 deletion, ranging in size from 1.25 to 3.25 Mb, 32 had a cfDNA result indicating a high probability of 22q11.2 deletion (sensitivity, 69.6% (95% CI, 55.2–80.9%)). All 689 maternal plasma samples without a 22q11.2 deletion were classified correctly by the cfDNA test as having no evidence of a 22q11.2 deletion (specificity, 100% (95% CI, 99.5–100%)).

**Conclusions:**

The results of this large‐scale prospective clinical evaluation of the sensitivity and specificity of a targeted cfDNA test for fetal 22q11.2 deletion demonstrate that this test can detect the common and smaller, nested 22q11.2 deletions with a low (0–0.5%) false‐positive rate. Although the positive predictive value (PPV) observed in this study population was 100%, the expected PPV in the general pregnant population is estimated to be 12.2% at 99.5% specificity and 41.1% at 99.9% specificity. The use of this cfDNA test to screen for 22q11.2 deletion could enhance identification of pregnancies at risk for 22q11.2 deletion syndrome without significantly increasing the likelihood of maternal anxiety and unnecessary invasive procedures related to a false‐positive result. © 2021 The Authors. *Ultrasound in Obstetrics & Gynecology* published by John Wiley & Sons Ltd on behalf of International Society of Ultrasound in Obstetrics and Gynecology.

## Abstract 2

Linked article: There is a comment on this article by Jing and Li. Click here to view the Correspondence.

**CONTRIBUTION**

*What are the novel findings of this work?*

This was a large‐scale prospective clinical evaluation of the sensitivity and specificity of a targeted cell‐free DNA test for fetal 22q11.2 deletion. In 735 pregnancies, including 46 with a 22q11.2 deletion, the cell‐free DNA test identified correctly 69.6% of affected cases as having a high probability of 22q11.2 deletion, with no false‐positive results.

*What are the clinical implications of this work?*

As the most common microdeletion, 22q11.2 deletion is associated with significant morbidity and mortality; however, its widely variable clinical features make it challenging to identify. Routine implementation of this targeted cell‐free DNA test could improve detection of fetal 22q11.2 deletion and guide pregnancy management without increasing the likelihood of a false‐positive result.

## INTRODUCTION

Deletions in the 22q11.2 chromosomal region are the most common microdeletion. They vary in size and can lead to a wide spectrum of clinical features known as 22q11.2 deletion syndrome^1^, ^2^. Of 22q11.2 deletion syndrome cases, 85% are caused by a 3‐Mb (specifically, 2.54‐Mb) deletion (i.e. the ‘common’ 22q11.2 deletion), and about 15% are due to smaller, nested deletions within the same region^3^. With a prevalence of about 1 in 1000 pregnancies, 22q11.2 deletions are the second most common genetic cause of congenital heart disease and developmental delay after Down syndrome^4^, ^5^, ^6^.

Prenatal screening for 22q11.2 deletion has potentially high clinical value because it can influence pregnancy management. 22q11.2 deletion syndrome is associated with morbidity and premature mortality, but the extreme variability in its clinical presentation can delay diagnosis for years after its features are observed^7^, ^8^. The corresponding missed opportunities for early intervention, anticipatory care and access to services can increase the likelihood of premature mortality as early as the neonatal period^9^, ^10^. Identification of a pregnancy at risk for 22q11.2 deletion could direct care towards detailed ultrasound evaluation and diagnostic testing in an effort to improve outcome^1^, ^11^, ^12^.

The ability of cell‐free DNA (cfDNA) analysis to detect fetal 22q11.2 deletion as early as the first trimester of pregnancy has been demonstrated in analytical validation studies and small cohorts^13^, ^14^, ^15^, ^16^, ^17^. However, professional societies have been reluctant to recommend such screening because the clinical performance of cfDNA analysis for 22q11.2 deletion requires further investigation^18^, ^19^, ^20^. To date, no prospective, large clinical validation study of prenatal cfDNA analysis for 22q11.2 deletion has been published.

The objective of this study was to assess the clinical performance of a targeted cfDNA test in screening for fetal 22q11.2 deletion, using blinded analysis of prospectively enrolled pregnancies with a fetal cardiac abnormality and stored clinical samples. Sensitivity and specificity were determined by comparing cfDNA results with fetal genetic status as determined by the current gold standard genetic diagnostic testing of samples obtained from chorionic villus sampling, amniocentesis or buccal swabs or of cord blood specimens.

## METHODS

As the prenatal prevalence of 22q11.2 deletion is 1 in 1000 pregnancies, a prohibitively large number of pregnancies would be needed in order to use a general clinical population to accurately evaluate cfDNA test performance for 22q11.2 deletion. An alternative approach, involving clinical samples with an increased likelihood of a 22q11.2 deletion, was used in this study in order to enable evaluation of test performance in a meaningful sample size, and as the test characteristics sensitivity and specificity are independent of prevalence. The final study population consisted of two groups: prospectively enrolled pregnancies with a fetal cardiac abnormality and clinical samples collected from a research biobank.

### Pregnancies enrolled prospectively

Between June 2015 and July 2019, 13 centers in Australia, Belgium, Germany, Italy, the USA and Taiwan enrolled pregnancies with a fetal cardiac abnormality as part of the Non‐Invasive Chromosomal Evaluation of 22q11.2 (22Q) study (NCT02541058). These pregnancies received genetic diagnostic testing for 22q11.2 deletion during the prenatal or neonatal period as the standard of care. Inclusion criteria were singleton gestation at ≥ 10 weeks and maternal age ≥ 18 years of age at the time of enrolment. The cut‐off date for enrolment was 26 July 2019.

Maternal blood samples were collected in Roche cfDNA collection tubes (Roche Diagnostics, San Jose, CA, USA) and sent to the Ariosa Diagnostics Inc. Clinical Laboratory Improvement Amendments‐certified laboratory for the Harmony® prenatal test.

The participating centers collected pregnancy characteristics, such as maternal age, gestational age at the time of blood collection, ultrasound findings, use of *in‐vitro* fertilization and egg donor status. The centers coordinated genetic diagnostic testing for every pregnancy according to local standards, using chromosomal microarray analysis, karyotyping with fluorescence *in‐situ* hybridization targeted to the 22q11.2 region and/or a comparable test (for example, quantitative fluorescence polymerase chain reaction or BACs‐on‐BEADS) of chorionic villi, amniocytes, cord blood or buccal swab.

All enrolled women provided written informed consent under the clinical study protocol (AD‐202). The protocol was conducted according to International Council for Harmonisation and Good Clinical Practice guidelines and was reviewed and approved by the ethics committee and/or the institutional review board of each participating center.

### Stored clinical samples

Plasma samples from pregnant women with confirmed fetal genetic status for the presence or absence of 22q11.2 deletion were received from a sample bank created as part of the RAPID non‐invasive prenatal testing (NIPT) evaluation study (RP‐PG‐0707‐10107), with national research ethics approval (13/LO/0082).

Maternal age and gestational age at the time of blood collection were provided. All participants had given written consent for their samples to be used for future research. Blood samples were collected prospectively in either Streck or ethylenediaminetetraacetic acid tubes and were spun twice, with plasma stored at –80°C.

### Sample processing

Samples were processed for the Harmony prenatal test with the addition of 22q11.2 DANSR (Digital Analysis of Selected Regions) assays and analyzed in a single custom microarray^14^. For each sample, a probability score incorporating the fetal fraction was generated by the fetal fraction optimized FORTE algorithm for 22q11.2 deletion^14^. Samples with a probability score of 1% or greater for a 22q11.2 deletion were classified as ‘high probability’, which could be fetal, maternal or both. Otherwise, the sample was classified as having no evidence of 22q11.2 deletion. Operators were blinded to fetal genetic status, defined as the 22q11.2 copy‐number status assessed by diagnostic testing. Results of cfDNA analysis were not communicated to study participants, since diagnostic test results were already available.

### Statistical analysis

Sensitivity and specificity of the cfDNA test for 22q11.2 deletion were calculated based on concordance between the cfDNA result and genetic status. Pregnancies with a chromosomal abnormality other than 22q11.2 deletion syndrome, such as trisomy, were classified as deletion negative. All CIs were determined using the Wilson method, using R statistical software package. *P*‐values < 0.05 were considered significant.

## RESULTS

### Characteristics of study population

The characteristics of both the prospectively enrolled pregnancies with a fetal cardiac abnormality and the stored clinical samples are shown in Table 1. Maternal plasma samples were collected from 370 prospectively enrolled pregnancies. Four cases did not meet the inclusion criteria and eight samples did not yield a cfDNA result. Therefore, the final prospectively enrolled study group consisted of 358 cases, of which 34 had a fetal 22q11.2 deletion; eight of these deletions were smaller than 2.5 Mb. Seventy‐eight cases had other findings, including whole‐chromosome aneuploidy and subchromosomal imbalance. The majority of the prospectively enrolled pregnancies had chromosomal microarray analysis (88%), and amniocentesis was the most common method of sampling for diagnostic testing (83%). Eight of the pregnancies were conceived by *in‐vitro* fertilization. A total of 377 stored clinical samples were included; data for 217 of these have been reported previously^14^. Twelve of the stored clinical samples were from pregnancies with a fetal 22q11.2 deletion, including one smaller deletion of 1.4 Mb. Combining the samples from the prospectively enrolled pregnancies with the stored clinical samples yielded a total of 735 maternal plasma samples that were eligible for analysis, including 46 with a fetal 22q11.2 deletion, ranging in size from 1.25 to 3.25 Mb.

**Table 1:** Characteristicsof the study population of 358 prospectively enrolled pregnancies with a fetal cardiac abnormality and 377 stored clinical samples, according to whether the pregnancy had 22q11.2 deletion (del)

	Prospectively enrolled pregnancies		Stored clinical samples	
Characteristic	22q11.2 del (*n* = 34)	No 22q11.2 del (*n* = 324)	22q11.2 del (*n* = 12)	No 22q11.2 del (*n* = 365)
Maternal age (years)	33 ± 5 (22–43)	31 ± 6 (16–47)	29 ± 5 (18–37)	32 ± 5 (19–47)
Gestational age (weeks)	24.3 ± 6.1 (13.9–36.0)	24.7 ± 6.3 (11.4–41.0)	20.9 ± 6.6 (10.0–34.1)	17.3 ± 5.7 (10.3–37.3)
Fetal fraction (%)	16.8 ± 8.6 (6.3–36.7)	17.2 ± 7.5 (5.1–41.8)	14.8 ± 6.4 (7.5–26.1)	14.4 ± 5.8 (5.2–43.0)

Data are given as mean ± SD (range).

### Screening performance

The performance of the cfDNA test for 22q11.2 deletion is presented in Table 2. Twenty‐four of the 34 prospectively enrolled pregnancies with fetal 22q11.2 deletion and eight of the 12 stored clinical samples with fetal 22q11.2 deletion were determined by cfDNA analysis to have a high probability of a 22q11.2 deletion. Therefore, in total, 32 of 46 samples were identified correctly by cfDNA analysis as having a high probability of 22q11.2 deletion (sensitivity, 69.6% (95% CI, 55.2–80.9%)). Six of those that were not detected were a smaller deletion of less than 2.5 Mb. The low number of cases with a smaller deletion precluded meaningful analysis of sensitivity according to deletion size. There was no significant difference in sensitivity between the prospectively enrolled pregnancies (70.6%) and the stored clinical samples (66.7%).

**Table 2:** Screening performance of a targeted cell‐free DNA test for 22q11.2 deletion in 735 maternal plasma samples, overall and separately in prospectively enrolled pregnancies with a fetal cardiac abnormality and in stored clinical samples

Variable	Prospectively enrolled pregnancies (*n* = 358)	Stored clinical samples (*n* = 377)*	Overall (*n* = 735)
Sensitivity	24/34 (70.6 (53.8–83.2))	8/12 (66.7 (39.0–86.2))	32/46 (69.6 (55.2–80.9))
Specificity	324/324 (100 (98.8–100))	365/365 (100 (99.0–100))	689/689 (100 (99.5–100))

Data are presented as *n*/*N* (% (95% CI)).

* Data for 217 of the stored clinical samples have been reported previously by Schmid *et al*.^14^.

The 689 maternal plasma samples from cases without a fetal 22q11.2 deletion comprised 324 prospectively enrolled pregnancies and 365 stored clinical samples. There were no false‐positive results; all cases without a fetal 22q11.2 deletion were classified correctly by cfDNA analysis as having no evidence of a 22q11.2 deletion (specificity, 100% (95% CI, 99.5–100%)).

## DISCUSSION

### Principal findings

In this study, 735 maternal plasma samples were evaluated by cfDNA analysis to determine the probability of a common or smaller, nested fetal 22q11.2 deletion. cfDNA results were compared with fetal genetic status based on diagnostic testing, which established a sensitivity of 69.6% (95% CI, 55.2–80.9%) and a specificity of 100% (95% CI, 99.5–100%). Notably, no false‐positive results were observed in this large‐scale prospective clinical evaluation of cfDNA test sensitivity and specificity for fetal 22q11.2 deletion.

### Interpretation of findings

The feasibility of cfDNA screening for 22q11.2 deletion was initially supported by studies demonstrating the technical ability of cfDNA analysis to detect deletions in laboratory‐generated plasma mixtures^13^, ^14^, ^15^. To date, most clinical studies have evaluated either very small numbers of affected pregnancies or larger datasets without complete outcome information^13^, ^14^, ^15^, ^16^, ^17^, ^21^, ^22^, ^23^, ^24^, ^25^, ^26^, ^27^.

Ravi *et al*.^16^ described the performance of a targeted single‐nucleotide polymorphism‐based cfDNA test in a retrospective cohort study of 400 clinical samples with confirmed genetic status for 22q11.2 deletion. One false‐positive result was identified, resulting in a specificity of 99.74%. Only 10 maternal plasma samples from pregnancies with 22q11.2 deletion were analyzed, all of which had the larger common deletion. Sensitivity was estimated to be 78.3% after adjusting for the exclusion of nested 22q11.2 deletions, but with a wide CI (95% CI, 50–89.8%) owing to the small sample size. Liang *et al*.^26^ reported > 99.9% specificity and 86.7% sensitivity for 22q11.2 deletion using a ‘genome‐wide’ next‐generation sequencing assay in a study of more than 90 000 women. Clinical follow‐up was obtained for 13 cases with a positive result; however, the lack of genetic outcome data for screen‐negative cases precluded the accurate calculation of sensitivity. The lower‐than‐expected frequency of 22q11.2 deletion found in their study population (< 1 in 7000 pregnancies compared with the expected frequency of 1 in 1000 pregnancies) suggests that many cases were not detected and the clinical sensitivity was overestimated.

For the targeted cfDNA test used in the current study, Schmid *et al*.^14^ reported previously an analytical sensitivity of 75.4% (95% CI, 67.1–82.2%). Specificity was determined to be at least 99.5% (95% CI, 99.0–99.7%) based on a clinical group of 1614 samples presumed to be unaffected. A recent prospective study by Kagan *et al*.^28^ used the test in 1127 pregnancies. Three false‐positive results were identified, corresponding to a specificity of 99.7%. The study was not intended to calculate sensitivity.

Collection of genetic outcome for every case in the current study enabled evaluation of both clinical sensitivity and clinical specificity. The 100% specificity and 70% sensitivity observed are consistent with the findings of previous studies of this targeted cfDNA test for 22q11.2 deletion^14^, ^28^ and were established in a true clinical population that included both common and smaller 22q11.2 deletions.

Fetal 22q11.2 deletions can manifest a variety of features, ranging in severity from subtle to severe, of which only some are identifiable on second‐trimester ultrasound^11^, ^12^. Even in the absence of physical malformations, 22q11.2 deletions are associated with an increased risk for morbidity and mortality as early as the neonatal period^7^, ^8^. The widely variable clinical expression of 22q11.2 deletion, clinicians' general lack of familiarity with the condition and the lack of an established pre‐ or postnatal screening protocol contribute to diagnostic delays even when clinical signs are present^12^, ^29^. The introduction of cfDNA analysis for prenatal 22q11.2 deletion screening in the first trimester could promote timely diagnosis, inform pregnancy management and enable early interventions aimed at improving outcomes.

Our results demonstrate the ability of a targeted cfDNA test to provide prenatal screening for common and nested 22q11.2 deletions, with a very low false‐positive rate (0–0.5%). While the established sensitivity, specificity and corresponding false‐positive rate are features of the test that are independent of population characteristics, the positive (PPV) and negative (NPV) predictive values are influenced by the prevalence of 22q11.2 deletion in the population studied. In our population, in which the incidence of 22q11.2 deletion was high (6.3%), the PPV was 100% and the NPV was 98%. In the general pregnant population, in which the prevalence of 22q11.2 deletion is estimated to be 1 in 1000, the expected PPV is estimated to be 12.2% at 99.5% specificity and 41.1% at 99.9% specificity, while the expected NPV is estimated to be > 99.9%.

Pregnancies with a cardiac abnormality are at increased risk for a variety of genomic imbalances, in addition to 22q11.2 deletion syndrome, that cfDNA testing does not detect. Although such high‐risk pregnancies were used in this study to establish a meaningful sample size, definitive diagnostic testing with chromosomal microarray analysis is the recommended approach in these cases^30^, ^31^, ^32^, ^33^. For patients who decline prenatal diagnosis after the identification of a fetal cardiac abnormality, cfDNA analysis could be helpful, as a high‐probability result would make a diagnosis of 22q11.2 deletion likely. However, a low‐probability result would be less useful.

### Strengths and limitations

The strengths of this study include the prospective collection of data from a large number of pregnancies with fetal 22q11.2 deletion, inclusion of both common and smaller 22q11.2 deletions and the availability of a genetic study for every pregnancy assessed. Multicenter prospective pregnancy enrolment enabled determination of the test performance for both the common and smaller, nested 22q11.2 deletions in a true clinical population. In total, 46 pregnancies with a 22q11.2 deletion were collected and studied, which is a 5‐fold higher number of affected cases than that in the next largest study reporting the sensitivity of cfDNA analysis for 22q11.2 deletion^16^. This study was limited to a single targeted cfDNA test and does not necessarily represent the performance of other targeted or non‐targeted cfDNA analysis methodologies.

### Conclusions

Prenatal screening for 22q11.2 deletion has become clinically feasible, but there have been limited studies in clinical populations. This study has established the sensitivity and specificity of a targeted cfDNA test for fetal 22q11.2 deletion in a large clinical population, which included both the common and smaller, nested deletions causing 22q11.2 deletion syndrome. The use of this test in the first trimester to screen for 22q11.2 deletion in the general pregnant population could be considered in an effort to improve early detection without significantly increasing the likelihood of a false‐positive result. Adoption into clinical care could be further supported by studies evaluating the PPV and NPV of the test in a general obstetric population.

## Data Availability

The data that support the findings of this study are available on request from the corresponding author. The data are not publicly available due to privacy or ethical restrictions.
